# Dr. Alfred I. Sawyer

**Published:** 1891-06

**Authors:** 


Dr. Alfred Isaac Sawyer, one of the most prominent homœopathic physicians in the State of Indiana, died at 6.45 o'clock, May 7th, at Monroe, Indiana, of apoplexy, in the sixty-third year of his age.

Dr. Sawyer was born in Lyme, Huron County, O., October 31st, 1828, and was the eleventh child and eighth son of a family of thirteen children. He studied medicine at the Western College of Homœopathy, at Cleveland, receiving his diploma in 1854. He then attended the New York University, fitting himself for the practice of ophthalmic surgery. He came to Monroe in May, 1857, and had resided there ever since. He was a Mason of high degree, and was for several years president of the Order of High Priesthood in the State. He was elected Mayor of Monroe in 1869, 1870, and 1877. This was the only office he ever held, but he was a Tilden elector in 1876. He was married June 21st, 1859, to Sarah Gazena Toll, who survives him, and they have two living children, a son and a daughter.

